# Supplementary material for: Isotopic evidence for an intensified hydrological cycle in the Indian sector of the Southern Ocean
Source: Nat Commun. 2023 May 13;14:2763. doi: 10.1038/s41467-023-38425-5 (PMC10183002; doi:10.1038/s41467-023-38425-5)
Supplement: Supplementary file 1 — Supplementary Information [file 41467_2023_38425_MOESM1_ESM.pdf]

Supplementary Information :

Isotopic evidence for an intensified hydrological cycle in the Indian sector of the Southern Ocean

Camille Hayatte Akhoudas[1,2*], Jean-Baptiste Sallée[3], Gilles Reverdin[3], F. Alexander Haumann[4,5,6], Etienne Pauthenet[7], Christopher C. Chapman[8], Félix Margirier[9], Claire Lo Monaco[3], Nicolas Metzl[3], Julie Meilland[10], and Christian Stranne[1,2]

[1]Department of Geological Sciences, Stockholm University, Stockholm, Sweden

[2]Bolin Centre for Climate Research, Stockholm University, Stockholm, Sweden

[3]CNRS/IRD/MNHN, LOCEAN, Sorbonne Université, Paris, France

[4]Alfred Wegener Institute, Helmholtz Centre for Polar and Marine Research, Bremerhaven, Germany

[5]Ludwig-Maximilians-University Munich, Munich, Germany

[6]Atmospheric and Oceanic Sciences Program, Princeton University, Princeton, United States

[7]LOPS, CNRS/IFREMER/IRD/UBO, Institut Universitaire Européen de la Mer, Plouzané, France

[8]CSIRO Environment, Earth Systems Science Program, Hobart, Tasmania, Australia

[9]School of Earth and Atmospheric Sciences, Georgia Institute of Technology, Atlanta, United States

[10]MARUM, University of Bremen, Bremen, Germany

Supplementary Information

Accompanying the article "Isotopic evidence for an intensified hydrological cycle in the Indian sector of the Southern Ocean" by Akhoudas, CH., Sallée, JB., Reverdin G., Haumann, FA., Pauthenet E., Chapman CC., Margirier F., Lo Monaco C., Metzl N., Meilland J. and Stranne C.

In this Supplementary Information file, we present several methods and figures in support of the main article text.

Salinity trends in the Indian sector of the Southern Ocean

In order to evaluate the described long-term hydrographic changes in this study, the computed zonal-mean salinity trend from our limited dataset between 40–90°E and 30–60°S is compared to supplementary salinity data in the region. All publicly available surface data between 1970 and 2018 were used and these amount to a total of around 18.5 thousand observations. Supplementary Figure 2 shows the computed zonal-mean salinity trend in surface waters from our dataset and in addition the computed zonal-mean salinity trend in the mixed layer from the supplementary data displayed by the blue dashed line. The mixed layer salinity trend is computed from three distinct types of observations in order to maximize spatial and temporal coverage. First, CTD profiles obtained from ship campaigns during the period 1970–2018. We use the NOAA World Ocean Database (https://www.nodc.noaa.gov/OC5/SELECT/dbsearch/dbsearch.html), and profiles obtained from the PANGAEA database (https://www.pangaea.de/). We only consider profiles that have a quality-control flag of 1, and that contain information on position, date and salinity[1]. This ship database is complemented by float data from the Argo international program (http://www.argo.ucsd.edu/) and, finally, profiles from marine mammal-borne sensors, obtained through the Marine Mammals Exploring the Oceans Pole to Pole program (http://www.meop.net/)[2]. This much larger dataset-based mixed layer salinity trend (blue dashed line on Supplementary Figure 2) displays two patterns coherent with what we observed from the surface salinity trend from our limited dataset; a region of surface salinification north of $\widetilde{lat}$ 46°S and a region of surface freshening south of $\widetilde{lat}$ 46°S. Using these observations comfort us on the robustness of the estimated salinity trends from our limited dataset and thus on the δ^{18}O trends which is concomitant with salinity observations.

Sea ice and P-E freshwater flux trends in the Southern Ocean

In this study, estimates of freshwater flux changes induced by sea ice come from Ref.[3]. Supplementary Figure 3 reveals large trends of net sea ice-ocean freshwater flux from the coast to the sea ice edge (1% sea ice concentration) over the period 1993–2008 in the whole circumpolar band, with regional dissimilarities. The zonal-mean sea ice freshwater flux trends are computed in the same streamwise coordinate system as for salinity and δ^{18}O computed trends (Supplementary Figure 4a). The trends show a region of increase sea ice melt north of $\widetilde{lat}$ 61°S and a sea ice melt decline further south (Supplementary Figure 4a). In the region of interest, between 40°E and 90°E, Supplementary Figure 4b shows a net sea ice melt decline from the coast to the sea ice edge over the period 1993–2008. The mean sea ice freshwater trend from the zonal-mean 40–90°E region corresponds to -31 $\pm$ 6 mm yr^{-1} per decade, i.e. tend to increase surface ocean salinity, with nevertheless local variability in the given region (Supplementary Figure 3). The mean sea ice freshwater flux trend over the latitude band we are considering as the subpolar sector in this study, between 60°S and 46°S, corresponds to -12 $\pm$ 4 mm yr^{-1} per decade.

Long-term P-E trends are highly uncertain, but can nevertheless be estimated from state-of-the-art atmospheric reanalysis

products (Supplementary Figure 5). We choose to investigate long-term trends in our region of interest (Table 1) and at the circumpolar scale from P-E time series between 1993 and 2021 from three global atmospheric reanalysis products: ERA5[4], JRA55[5] and GPCP coupled with OAFlux[6,7]. ERA5 and JRA55 consistently simulate the same spatial patterns and intensities and both differ from the GPCP/OAFlux trends. In addition to simulate different meridional structure in the Indian sector of the Southern Ocean (Supplementary Figure 6a) and at the circumpolar scale (Supplementary Figure 6b), they show large inconsistencies both in spatial patterns and intensities compared with the surface salinity trends. When compared with a long time series of precipitation from one meteorological station (https://www.iaea.org/services/networks/gnip) at Marion Island (46°54S–37°51E) from 1961 to 2013, the simulated precipitations are a factor 2 lower than observed, while the three reanalyses products are in good agreement (Supplementary Figure 6c), suggesting they might also underestimate P-E changes by a similar factor. To summarize, the different products show very poor capabilities and are not in agreement with the salinity trend patterns observed in the region. We consider that among the different freshwater forcings, changes in air-sea flux is by far the most poorly known from observations.

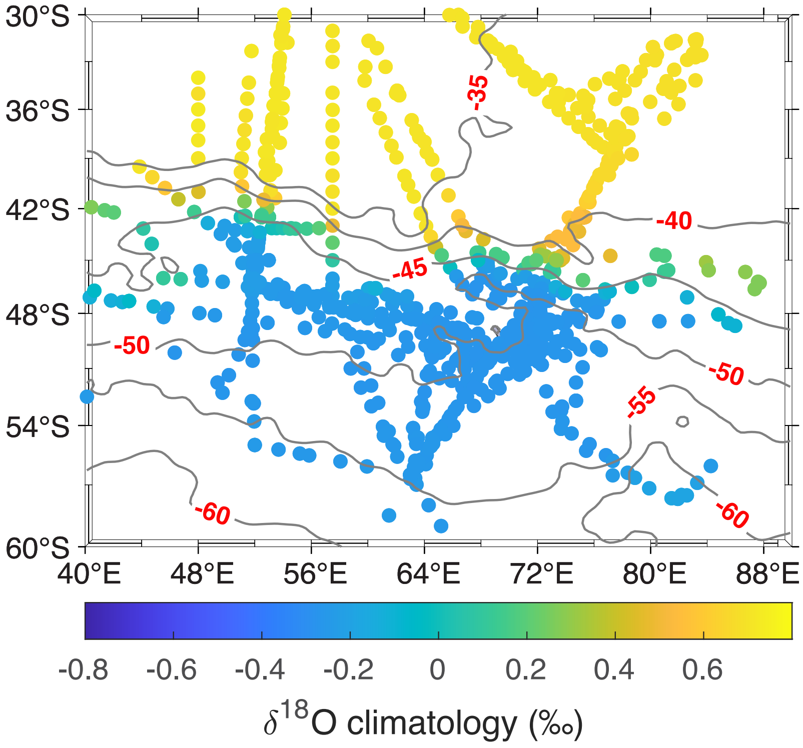

Supplementary Figure 1: Oxygen isotope climatology in the Indian sector of the Southern Ocean. The spatial variability in the isotopic composition of the surface ocean shown by the mean of all observations from 1993 to 2021. Positions of the $\widetilde{lat}$ contours are plotted as grey lines and labelled in red. The gradual depletion in δ^{18}O content of seawater from the subtropics toward high latitudes is understood as a result of the influence of depleted meteoric water because of progressive removal of oxygen 18 from the moist air masses being transported from the high evaporated tropical regions toward the poles.

Supplementary Table 1. Estimated values and their uncertainties of P-E flux trends in mm yr^{-1} per decade from 1993 to 2021, computed from reanalyses in the corresponding regions.

	North of $\widetilde{lat}$ 46°S (Subtropical)	South of $\widetilde{lat}$ 46°S (Subpolar)
ERA5	5 ± 9	10 ± 5
JRA55	-5 ± 12	4 ± 5
GPCP/OAFlux	60 ± 34	17 ± 30

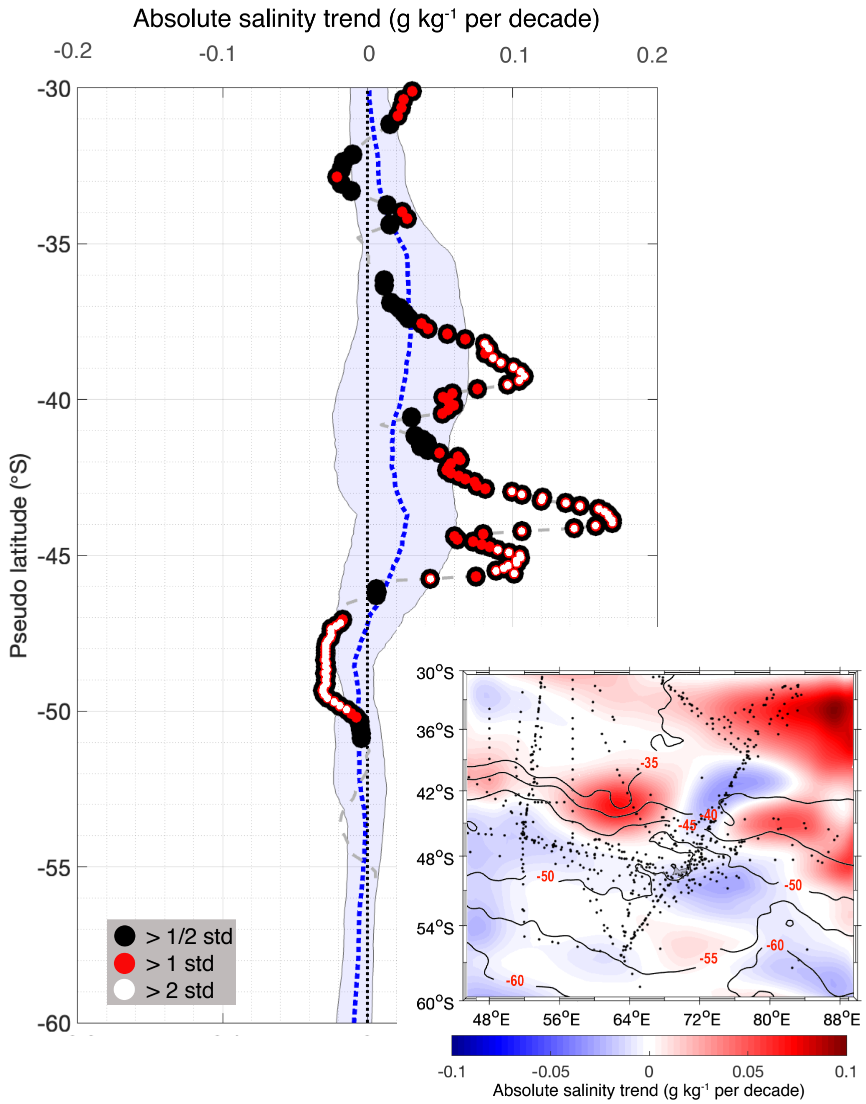

Supplementary Figure 2: Meridional profile and map of salinity trend. Zonal-mean meridional profile of salinity trends (grey dashed line) based on the *GISS/OISO* observations. The black dots represent the trends larger than half their estimated standard errors (> 1/2 std), the red dots the trends larger than their estimated standard errors (> 1 std) and white dots the trends larger than double their estimated standard errors (> 2 std). Trends are computed using an irregular mean dynamic topography grid with bin size ranging from 0.1 to 0.3 m. The blue dashed line and shading represent surface salinity trends and their associated errors based on a larger dataset spanning between 1970 and 2018[1]. In addition, the regional map of the surface salinity trend in color based on the larger dataset as mentioned above is shown in the bottom right panel. The black dots represent the location of the ship-based observations obtained between 1993 and 2021 from our study and position of the $\widetilde{lat}$ contours are plotted in black lines and labelled in red.

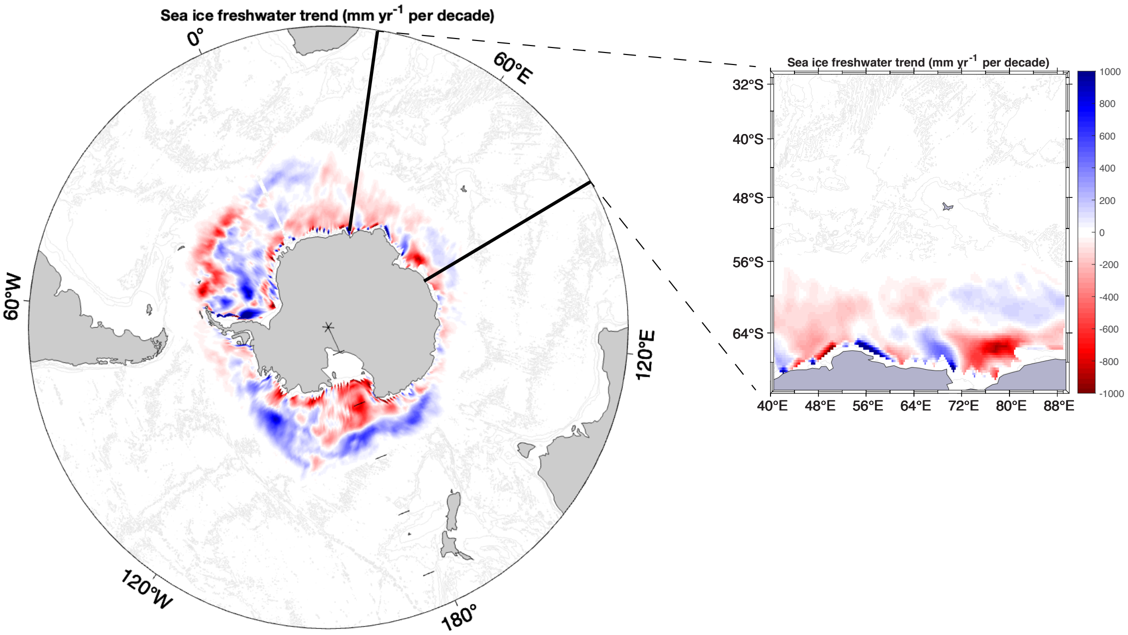

Supplementary Figure 3: Maps of sea ice freshwater trend. Southern Ocean map showing sea ice freshwater flux trends from Ref.[3] estimated between 1993 and 2008 using an irregular mean dynamic topography grid with bin size ranging from 0.1 to 0.3 m as for salinity and δ^{18}O computed means, anomalies, and associated trends.

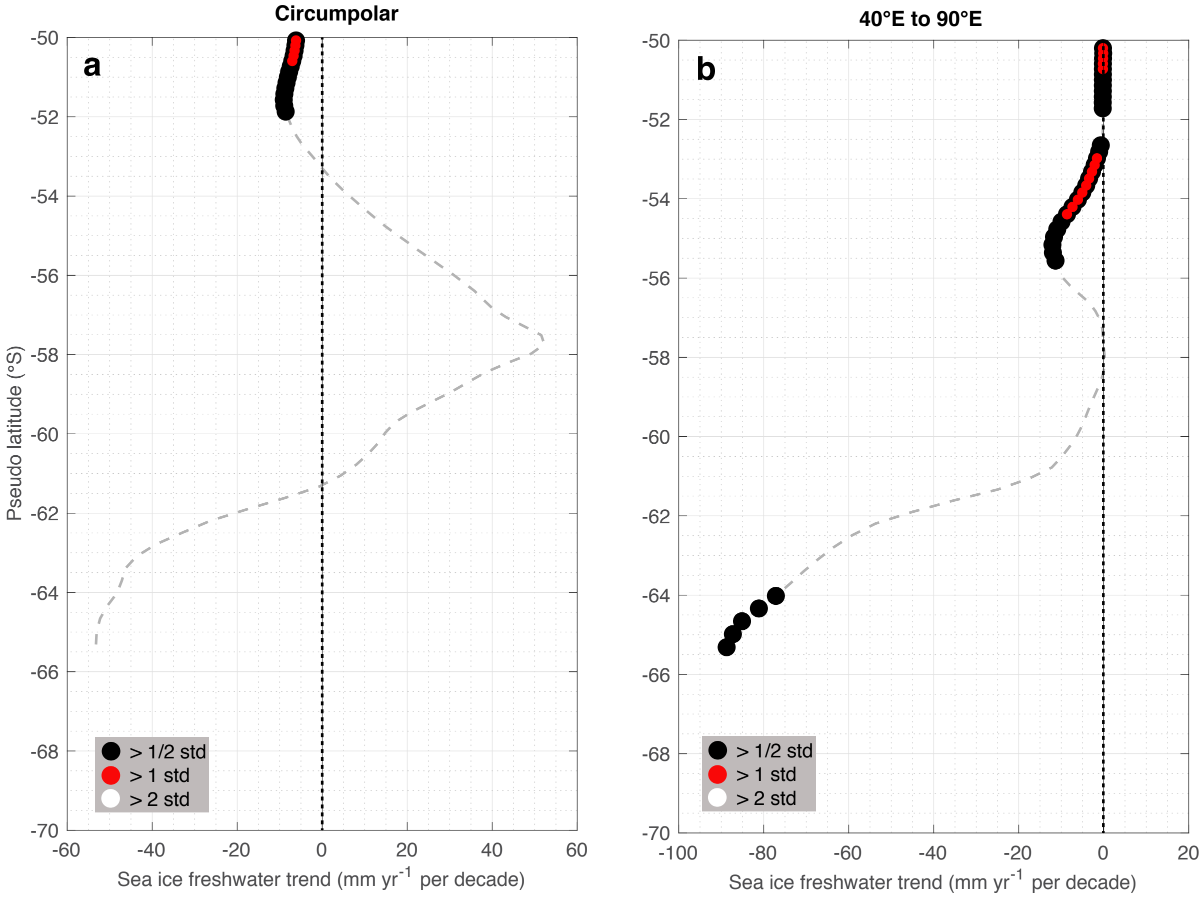

Supplementary Figure 4: Meridional profiles of sea ice freshwater trend. Zonal-mean sea ice trends (grey dashed line) based on Supplementary Figure 3 at the circumpolar scale (a) and in the region of interest between 40°E and 90°E in the Indian sector of the Southern Ocean (b). The black dots represent the trends larger than half their estimated standard errors (> 1/2 std), the red dots the trends larger than their estimated standard errors (> 1 std) and white dots the trends larger than double their estimated standard errors (> 2 std). Trends are computed using an irregular mean dynamic topography grid with bin size ranging from 0.1 to 0.3 m.

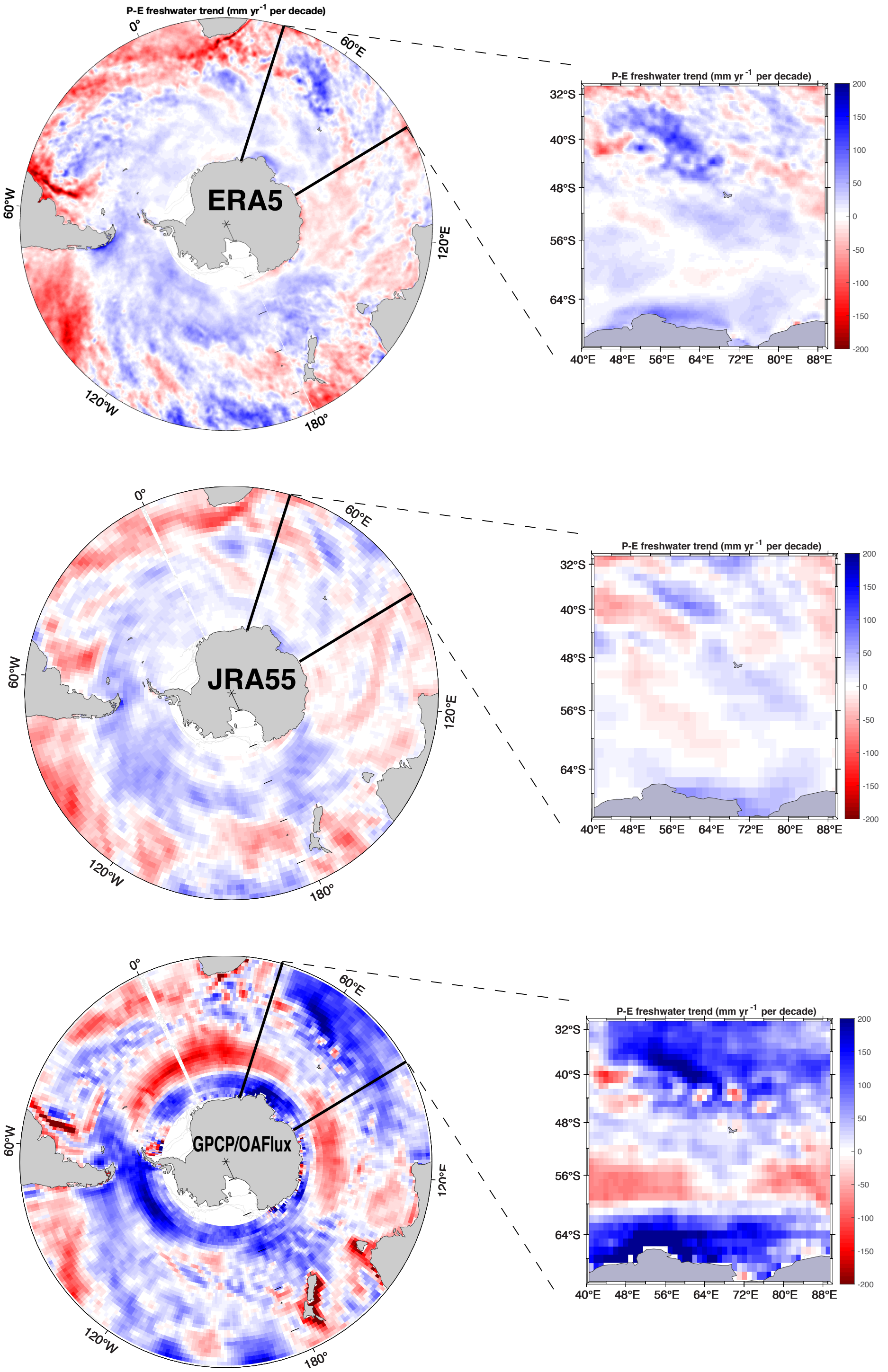

Supplementary Figure 5: Maps of net precipitation trend. Southern Ocean maps showing P-E trends from ERA5, JRA55 and GPCP coupled with OAFlux reanalysis products, estimated between 1993 and 2021 using an irregular mean dynamic topography grid with bin size ranging from 0.1 to 0.3 m as for salinity and δ^{18}O computed means, anomalies, and associated trends.

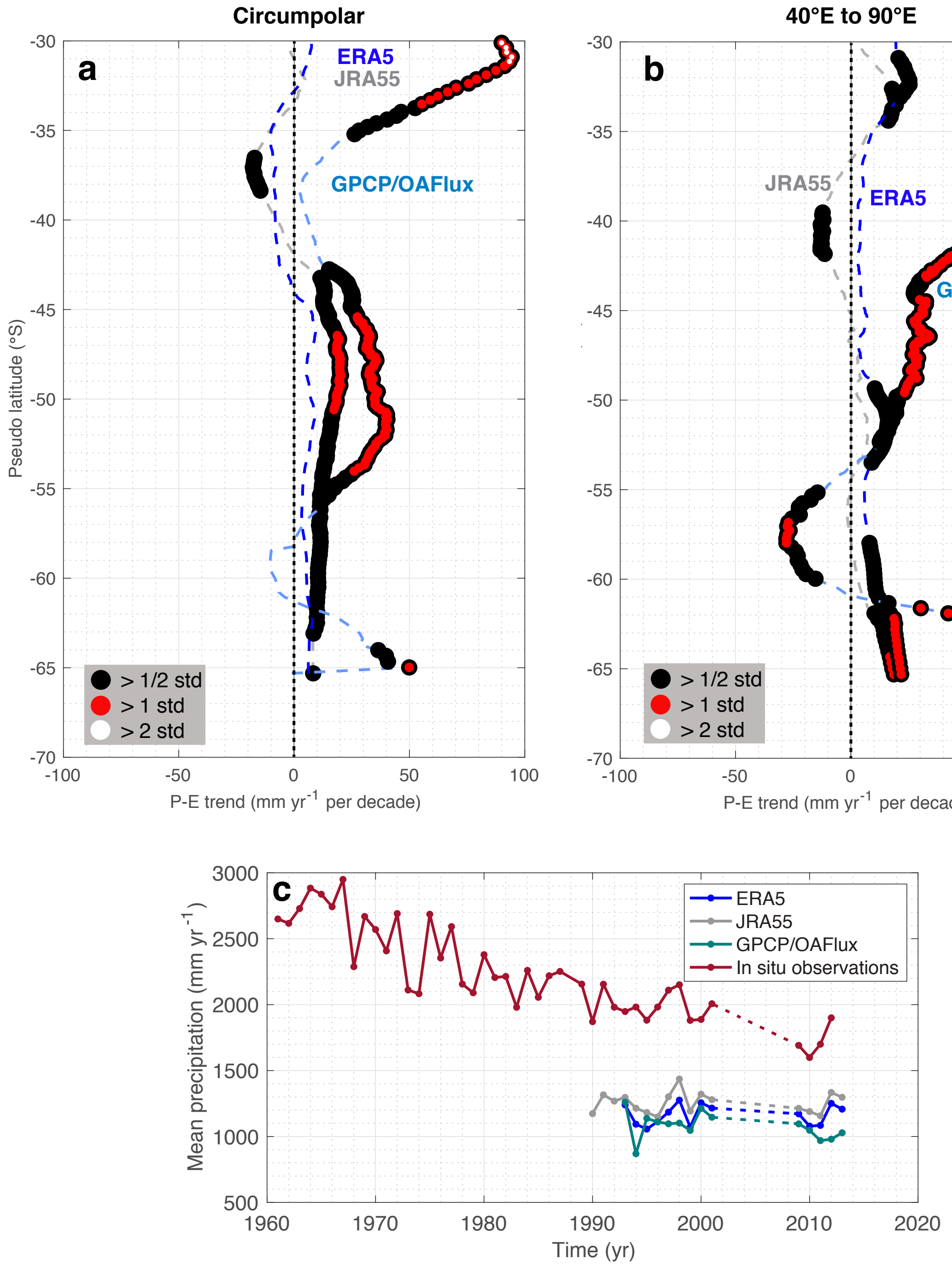

Supplementary Figure 6: Meridional profiles of net precipitation trend and time-series of mean precipitation. Zonal-mean meridional profile of P-E trends (grey dashed line) based on Supplementary Figure 5 at the circumpolar scale (a) and in the region of interest between 40°E and 90°E in the Indian sector of the Southern Ocean (b). The black dots represent the trends larger than half their estimated standard errors (> 1/2 std), the red dots the trends larger than their estimated standard errors (> 1 std) and white dots the trends larger than double their estimated standard errors (> 2 std). Trends are computed using an irregular mean dynamic topography grid with bin size ranging from 0.1 to 0.3 m. The panel (c) shows the mean precipitation at Marion Island (46°54S–37°51E) from 1961 to 2013 for *in situ* observations in red, ERA5 in blue, JRA55 in grey and GPCP/OAFlux in green.

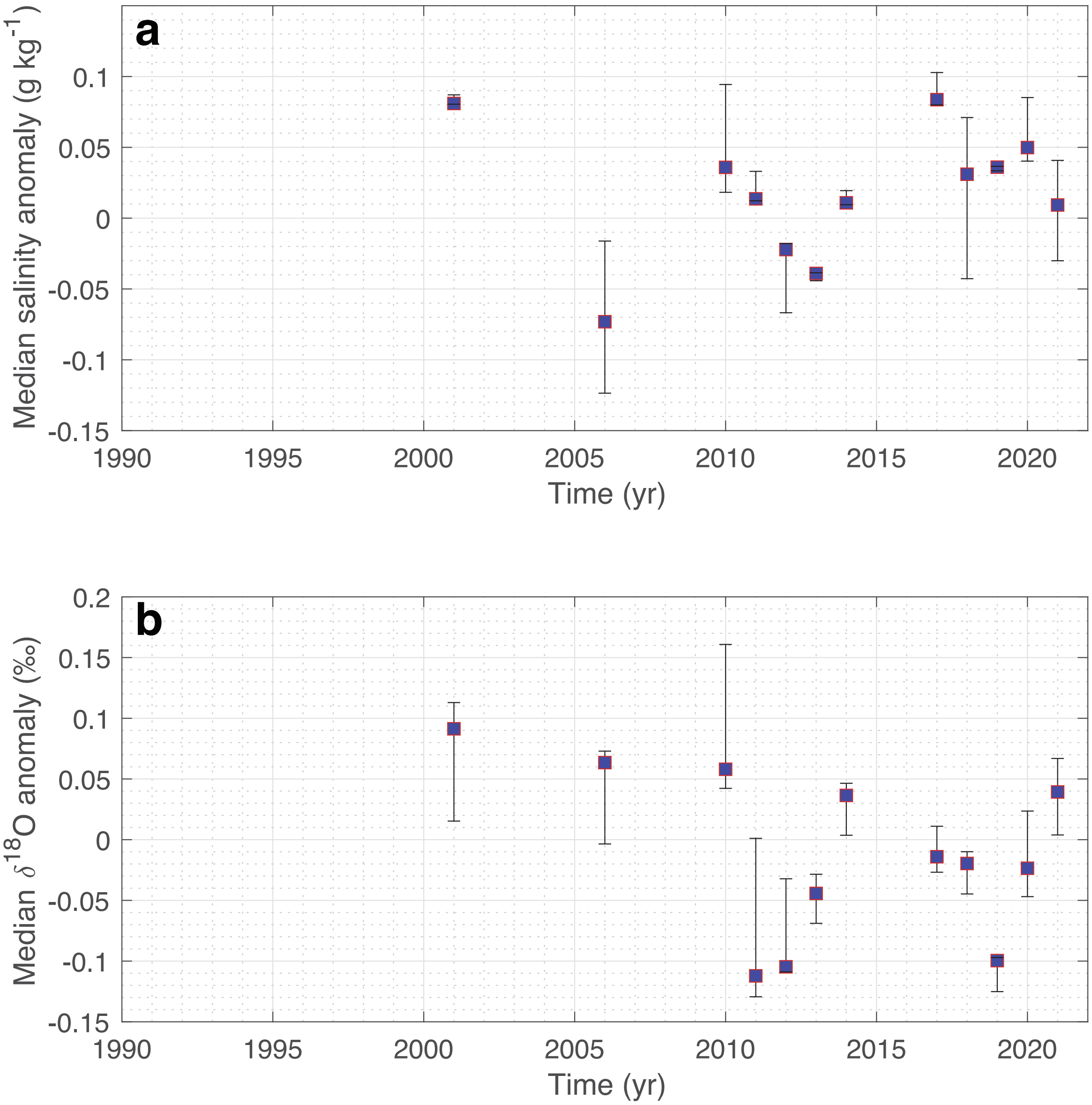

Supplementary Figure 7: Salinity and oxygen isotope anomalies time-series. Median salinity (a) and δ^{18}O (b) anomaly time series for the region south of $\widetilde{lat}$ 52°S. Each time series panel shows: the annual median anomaly (purple square from the regional salinity and δ^{18}O climatology), computed for each individual observation and error bars shown in black referring to the 25th–75th percentile range.

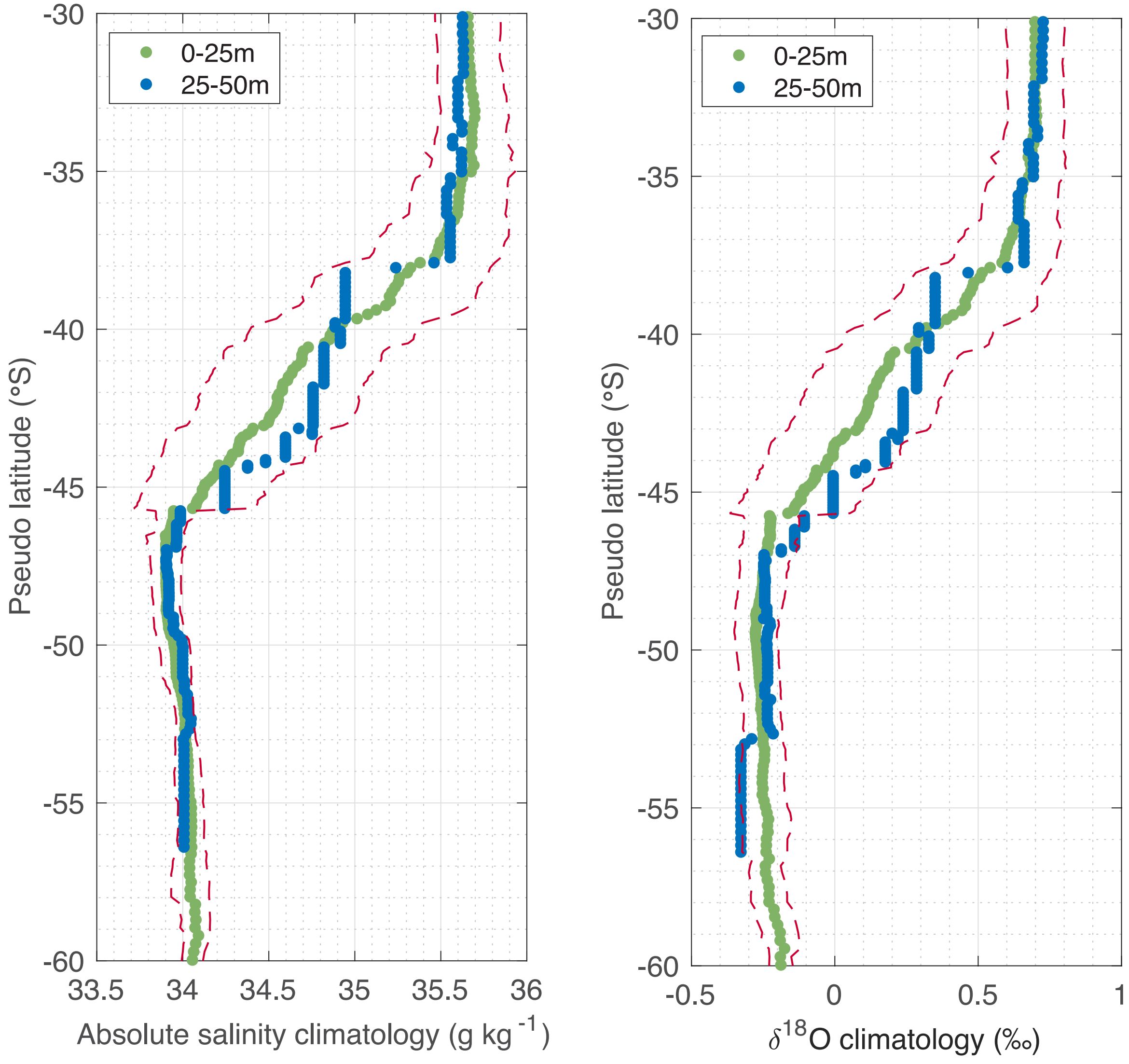

Supplementary Figure 8: Meridional profiles of salinity and oxygen isotope climatology. Meridional profiles of zonally averaged salinity (left) and δ^{18}O (right) and their standard errors in red dashed lines. Green dots are observations between 0- and 25-meters depth and blue dots are observations between 25- and 50-meters depth. Calculations are computed using an irregular mean dynamic topography grid with bin size ranging from 0.1 to 0.3 m.

Supplementary References

1. Sallée, J.-B. *et al.* Summertime increases in upper-ocean stratification and mixed-layer depth. *Nature* **591**, 592–598 (2021).
2. Treasure, A. M. *et al.* Marine mammals exploring the oceans pole to pole: a review of the meop consortium. *Oceanography* **30**, 132–138 (2017).
3. Haumann, F. A., Gruber, N., Münnich, M., Frenger, I. & Kern, S. Sea-ice transport driving southern ocean salinity and its recent trends. *Nature* **537**, 89–92 (2016).
4. Hersbach, H. *et al.* The era5 global reanalysis. *Q. J. Royal Meteorol. Soc.* **146**, 1999–2049 (2020).
5. Kobayashi, S. *et al.* The jra-55 reanalysis: General specifications and basic characteristics. *J. Meteorol. Soc. Jpn. Ser. II* **93**, 5–48 (2015).
6. Huffman, G. J. *et al.* Global precipitation at one-degree daily resolution from multisatellite observations. *J. hydrometeorology* **2**, 36–50 (2001).
7. Yu, L. & Weller, R. A. Objectively analyzed air–sea heat fluxes for the global ice-free oceans (1981–2005). *Bull. Am. Meteorol. Soc.* **88**, 527–540 (2007).
